# Oculocutaneous albinism in a patient with an *OCA2* variant: molecular and clinical insights

**DOI:** 10.2478/abm-2025-0019

**Published:** 2025-06-30

**Authors:** Mostafa Neissi, Sahar Kareem Al-Mozani, Ayoob Radhi Al-Zaalan, Samaneh Sanavi Shiri, Motahareh Sheikh-Hosseini, Adnan Issa Al-Badran, Elaheh Nekouei

**Affiliations:** Department of Genetics, Khuzestan Science and Research Branch, Islamic Azad University, Ahvaz 61349-37333, Iran; Department of Genetics, Ahvaz Branch, Islamic Azad University, Ahvaz 61349-37333, Iran; Noor-Gene Genetic Laboratory, Ahvaz, Iran; Department of Biology, University of Misan, Misan 62001, Iraq; Department of Medical Lab Technology, College of Health and Medical Technology, Southern Technical University, Basrah 61006, Iraq; Amiralmomenin Hospital, Gerash 74417-57897, Iran; Pediatric Cell and Gene Therapy Research Center, Tehran University of Medical Sciences, Tehran 14194, Iran; Department of Biology, College of Science, University of Basrah, Basrah 61004, Iraq; Department of Biomedical and Clinical Science, Linköping University, University Hospital, Campus US, Linköping SE-581 83, Sweden

**Keywords:** anexome-sequencing, genetic diagnosis, *OCA2* gene, oculocutaneous albinism, variant

## Abstract

**Background:**

Albinism is a rare genetic condition characterized by hypopigmentation of the skin, hair, and eyes, as well as visual impairments. Oculocutaneous albinism type 2 (*OCA2*) is commonly associated with variants in the *OCA2* gene, which encodes a protein critical for melanosomal pH regulation and melanin biosynthesis. Exome sequencing, validated by Sanger sequencing, was employed to investigate the genetic basis of albinism in a consanguineous Iranian family. Bioinformatics analyses and structural modeling were conducted to assess the pathogenicity and impact of the detected variant.

**Case presentation:**

A 27-year-old male from a consanguineous Iranian family presented with features of oculocutaneous albinism, including white hair, blue eyes, strabismus, sun-sensitive skin, reduced visual acuity, and significant photophobia, resulting in functional limitations in bright environments. Genetic analysis identified a novel homozygous missense variant in the *OCA2* gene, NM_000275.3:c.1274T>G (p.Met425Arg), located in exon 13. The genomic coordinates of the variant are chr15:g.27985154A>C (GRCh38/hg38). In silico tools classified the variant as likely pathogenic based on its evolutionary conservation, absence in population databases, and structural modeling predictions. Segregation analysis confirmed autosomal recessive inheritance, with both parents being heterozygous carriers.

**Conclusion:**

The identified *OCA2* variant, c.1274T>G; p.Met425Arg, disrupts protein function, impairing melanosomal activity and melanin biosynthesis. This study underscores the importance of genetic analysis in characterizing *OCA2* variants and highlights the need for further exploration of molecular mechanisms and phenotypic variability in *OCA2*-related albinism to improve diagnosis and counseling.

Albinism is a rare, genetically inherited condition characterized by the partial or complete loss of melanin pigment in the skin, hair, and eyes. This hypopigmentation often results in distinct physical traits and a range of medical challenges, including visual impairments such as nystagmus, reduced visual acuity, photophobia, and strabismus. Additionally, individuals with albinism are more susceptible to skin damage and ultraviolet-induced malignancies. Albinism is broadly classified into oculocutaneous albinism (OCA), which affects pigmentation in the skin, hair, and eyes, and ocular albinism (OA), which primarily affects the eyes without significant involvement of the skin and hair [1, 2].

The etiology of albinism involves variants in genes that regulate melanin biosynthesis, melanosomal function, or pigment cell signaling. Several genes have been implicated in its pathogenesis, including *TYR*, *TYRP1*, *SLC24A5*, *SLC45A2*, *MC1R*, and *ACP1*. Among these, the *OCA2* gene is exclusively responsible for OCA type 2 (OCA2), which is one of the most prevalent forms of albinism worldwide. The *OCA2* gene encodes the P protein, a critical regulator of melanosome pH, which is essential for optimal tyrosinase activity—the key enzyme in eumelanin biosynthesis, the predominant melanin pigment in human skin and hair [3,4,5,6,7,8].

Understanding the molecular basis of albinism has advanced significantly with insights into the melanogenesis pathway. This pathway is regulated by the microphthalmia-associated transcription factor (MITF), a master regulator of genes encoding key melanogenic enzymes and structural proteins. Variants in *OCA2* and related genes disrupt melanosome biogenesis, melanocyte function, and the enzymatic activity required for pigment synthesis [9].

Exome sequencing has emerged as a powerful and efficient tool for identifying genetic variants, particularly in rare disorders and cases involving consanguineous parents, where the likelihood of homozygous variants is significantly higher. Previous studies have demonstrated the efficacy of exome sequencing in detecting pathogenic variants associated with a wide range of inherited conditions, providing crucial insights into their molecular underpinnings [10,11,12,13]. Building on this evidence, in the present study, we employed exome sequencing, complemented by Sanger sequencing for validation, to investigate the genetic basis of albinism in an Iranian family affected by this condition.

## Case presentation

All procedures performed in this study adhered to the ethical standards of the institutional and/or national research committee and complied with the 1964 Declaration of Helsinki and its later amendments or comparable ethical standards. The patient was informed that the images would be published in a scientific journal and could be freely accessible online. Written informed consent was obtained from the patient for publication of the clinical details and accompanying images, including identifiable facial features.

### Clinical features and patient history

**Figure 1.**
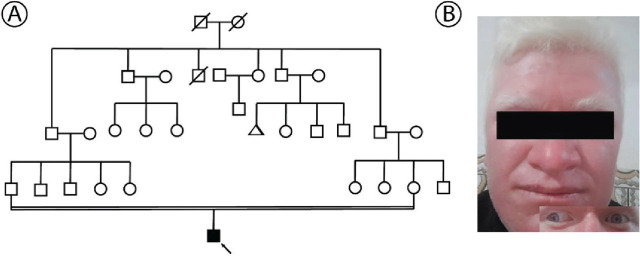
**(A)** Pedigree of the family with albinism. Squares represent male individuals, circles represent female individuals, the black square represents the affected male patient, slashes indicate deceased individuals, and the arrow denotes the proband. Consanguineous marriage is indicated by double lines, suggesting a recessive inheritance pattern. **(B)** The proband with albinism shows characteristic features, including white hair, blue eyes, and right-eye deviation indicative of strabismus. Published with the patient’s written informed consent.

A 27-year-old male with albinism, born to consanguineous parents from an Iranian family (**Figure 1A**), was referred to the Noor-Gene Genetic Laboratory in Ahvaz, Iran, for genetic analysis. The patient exhibits features of OCA, including white hair, blue eyes, and a deviation of the right eye suggestive of strabismus (**Figure 1B**). He also experiences visual impairment, including reduced visual acuity and significant photophobia, which are characteristic of albinism and contribute to functional limitations in bright environments. His skin is thinner than average, highly sensitive to sunlight, and prone to sunburn and photodamage. However, his nails, bones, and muscles are within normal parameters.

The patient’s immediate family, including his parents and sister, are unaffected by albinism. To manage his condition, he adopts precautionary measures such as wearing hats, sunglasses, and long-sleeved clothing to minimize sun exposure, particularly during summer months.

### Sample collection and genomic DNA extraction

Peripheral blood samples (5 mL) were collected from the affected individual and both parents in EDTA-containing tubes to prevent coagulation. Genomic DNA was extracted using the QIAamp DNA Mini Kit (Qiagen) according to the manufacturer’s protocol. The quality and concentration of the extracted DNA were assessed using a NanoDrop spectrophotometer (Thermo Fisher Scientific) and agarose gel electrophoresis. DNA samples were stored at −20°C for downstream applications.

### Exome sequencing

Exome sequencing was performed on the proband’s DNA to identify potential pathogenic variants. DNA enrichment for exonic regions was achieved using the Agilent SureSelect Human All Exon Kit V7 (Agilent Technologies). Sequencing was conducted on the Illumina NovaSeq 6000 platform (Illumina), generating 150 bp paired-end reads.

The sequencing data were processed using an established bioinformatics pipeline. Raw reads were assessed for quality using FastQC (https://www.bioinformatics.babraham.ac.uk/projects/fastqc/). High-quality reads were aligned to the GRCh38 human reference genome using the Burrows–Wheeler Aligner (BWA) MEM algorithm. Duplicate reads were marked using Picard (http://broadinstitute.github.io/picard/), and base quality score recalibration and variant calling were performed using the Genome Analysis Toolkit (GATK).

### Variant filtering and prioritization

Variants were annotated and filtered using ANNOVAR (http://annovar.openbioinformatics.org/) and variant effect predictor (VEP). Variants with minor allele frequencies (MAF) >0.01 in population databases such as gnomAD (https://gnomad.broadinstitute.org/) and 1,000 Genomes (https://www.internationalgenome.org/) were excluded. Candidate variants were further evaluated based on segregation analysis within the family. The novelty of variants was assessed using databases including HGMD (https://www.hgmd.cf.ac.uk/ac/index.php), ClinVar (https://www.ncbi.nlm.nih.gov/clinvar/), dbSNP (https://www.ncbi.nlm.nih.gov/snp/), and a literature review.

The pathogenic potential of the identified variant was assessed using various bioinformatics tools, including MutationTaster (http://www.mutationtaster.org), PolyPhen-2 (http://genetics.bwh.harvard.edu/pph2/), SIFT (https://sift.bii.a-star.edu.sg/), CADD (https://cadd.gs.washington.edu/), FATHMM (http://fathmm.biocompute.org.uk/), and others, ensuring a comprehensive evaluation of its potential impact.

### Protein structural and functional analysis

The location of the candidate variant within the protein was analyzed for conservation using Clustal Omega (https://www.ebi.ac.uk/Tools/msa/clustalo/) and NCBI’s Conserved Domain Database (https://www.ncbi.nlm.nih.gov/Structure/cdd). Structural modeling was performed using SWISS-MODEL (https://swissmodel.expasy.org/) to evaluate the impact of the variant on protein conformation.

### Protein–protein interaction analysis

To understand the functional relevance of the detected gene, protein–protein interaction (PPI) networks were constructed using the STRING database (https://string-db.org/). Functional enrichment analysis was performed to determine the biological pathways and processes associated with the identified gene.

### Polymerase chain reaction and Sanger sequencing

To validate the identified candidate variant, a specific primer set was designed to flank the target region (primer sequences available upon request). Polymerase chain reaction (PCR) amplification was performed using the Platinum™ SuperFi II PCR Master Mix (Thermo Fisher Scientific) in a thermocycler under optimized conditions: initial denaturation at 98°C for 2 min, followed by 35 cycles of denaturation at 98°C for 10 s, annealing at a primer-specific temperature for 20 s, and extension at 72°C for 30 s, with a final extension at 72°C for 5 min.

The PCR amplicons were purified using the QIAquick PCR Purification Kit (Qiagen) to remove excess primers and dNTPs. Sequencing was conducted using BigDye™ Terminator v3.1 Cycle Sequencing Kit (Applied Biosystems) according to the manufacturer’s protocol. The sequencing products were analyzed on an ABI 3500 Genetic Analyzer (Applied Biosystems), and the chromatograms were visualized and interpreted using Chromas software (Technelysium).

### Segregation analysis

Segregation of the candidate variant was assessed in the affected individual and both parents using the same PCR and Sanger sequencing protocol. The presence or absence of the variant was confirmed by analyzing the sequencing chromatograms to establish its inheritance pattern within the family.

## Results

### Identification of a novel OCA2 variant

Exome sequencing identified a novel missense variant in the *OCA2* gene, NM_000275.3:c.1274T>G (p.Met425Arg). According to the GRCh38 (hg38) reference genome, this variant corresponds to a genomic coordinate of chr15:g.27985154A>C. The nucleotide substitution from thymine (T) to guanine (G) at position 1274 is located in exon 13 of 24 and leads to an amino acid change from methionine (Met) to arginine (Arg) at position 425.

### In silico pathogenicity predictions

Bioinformatics analysis using multiple tools predicted the c.1274T>G (p.Met425Arg) variant to be likely pathogenic. Computational tools such as PolyPhen-2, PROVEAN, and CADD reported high pathogenicity scores, indicating a deleterious impact on protein function.

The chr15:g.27985154A>C variant showed consistent evidence of pathogenicity across various computational tools. PolyPhen-2 (HumDiv) classified the variant as “probably damaging” with a score of 0.999. AlphaMissense provided a pathogenic-supporting classification with a score of 0.9455. Both SIFT and SIFT4G predicted pathogenicity, with scores of 0.001 and 0.002, respectively. PROVEAN identified the variant as pathogenic with a score of −5, while CADD reported it as highly deleterious with a score of 27.7. FATHMM-MKL and FATHMM-XF gave pathogenic-supporting scores of 0.9854 and 0.9607, respectively.

DEOGEN2 and EIGEN yielded pathogenic-supporting scores of 0.8468 and 0.7316, with EIGEN PC providing a similar score of 0.7346. EVE classified the variant as pathogenic-supporting (score: 0.6185), and LRT confirmed pathogenicity with a score of 0. MutationTaster indicated a disease-causing effect with a perfect score of 0.9999. According to MutationTaster, protein features between amino acids 424 and 440 include a TRANSMEM domain described as Helical, which is lost.

Other tools such as BayesDel addAF (score: 0.4461) and BayesDel noAF (score: 0.4029) supported a pathogenic classification. Both REVEL and MetaLR categorized the variant as pathogenic, each with a score of 0.9365. MetaRNN provided a strong pathogenicity score of 0.986, while MetaSVM predicted pathogenicity with a score of 0.9365. Finally, MVP classified the variant as pathogenic moderate with a score of 0.9891, and MutPred assigned it a pathogenic strong (PS) classification with a score of 0.9.

According to the American College of Medical Genetics and Genomics (ACMG) and the Association for Molecular Pathology (AMP) guidelines, the PP3 criterion (strong pathogenicity) is fulfilled due to computational prediction tools unanimously supporting a deleterious effect on the gene. An aggregated pathogenicity prediction score of 0.982, which falls within the PS range (0.9–1.0), further supports this classification.

The PM2_supporting criterion is fulfilled by the variant’s absence in population databases such as gnomAD (0.0% frequency), consistent with ClinGen guidelines. Additionally, the PM3_supporting criterion is met based on confirmed heterozygous parental carrier status, which strengthens the evidence for homozygosity in the proband. Together with strong computational evidence (PP3) unanimously supporting a deleterious effect on the gene, these criteria classify the variant as “likely pathogenic” based on ACMG/AMP guidelines, incorporating one PS and two Pathogenic Supporting (PP) criteria.

### Protein structure analysis

**Figure 2.**
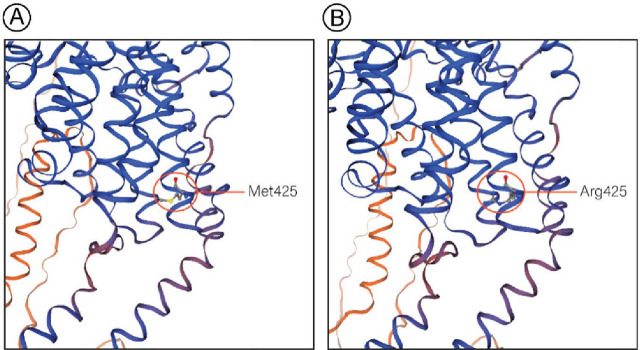
Predicted 3D structures of the OCA2 protein: (**A**) Wild type (Met425) and (**B**) mutant (Arg425) variants, modeled using the Swiss Model server. OCA2, oculocutaneous albinism type 2.

Structural modeling of the wild-type and mutant OCA2 protein (**Figure 2**) revealed that the replacement of methionine with arginine at position 425 alters the local chemical environment by introducing a positively charged residue. This substitution may affect the stability of the protein, its folding, or interactions with surrounding residues. Although the overall structure appears similar, these localized alterations could impair OCA2 function in melanin biosynthesis.

### Functional analysis

**Figure 3.**
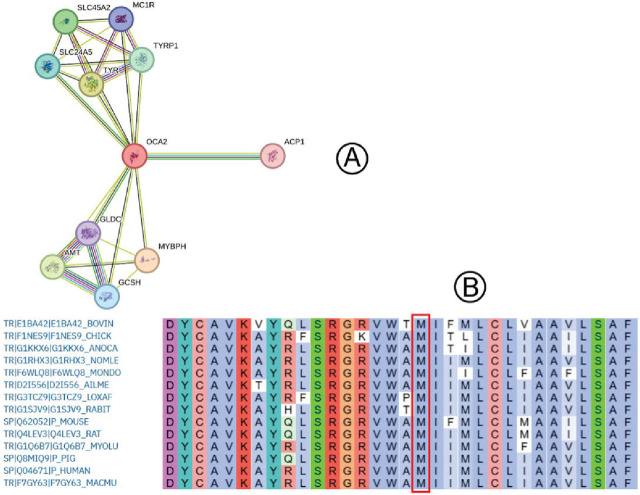
(**A**) PPI network analysis for the *OCA2* gene, illustrating interactions with genes involved in melanin synthesis and pigmentation processes. (**B**) Conservation analysis of the methionine residue at position 425 across 13 species, showing complete conservation, which highlights the functional importance of this amino acid in the OCA2 protein. OCA2, oculocutaneous albinism type 2; PPI, protein-protein interaction.

PPI analysis was performed to explore the functional relevance of the identified gene (**Figure 3A**). The analysis revealed interactions between the OCA2 protein and several key proteins involved in melanin synthesis and pigment formation, including TYR, TYRP1, SLC24A5, SLC24A2, MC1R, ACP1, GLDC, GCSH, and MYBPH. Functional enrichment analysis further highlighted the biological pathways and processes associated with OCA2, underscoring its role in pigmentation-related activities. Additionally, a conservation analysis (**Figure 3B**) demonstrated that the methionine residue at position 425 is highly conserved across 13 species. The alignment shows complete conservation at this position, emphasizing its functional importance in the OCA2 protein. Such conservation supports the hypothesis that alterations at Met425 could disrupt critical biological functions related to melanin synthesis and pigment formation.

### Validation and segregation analysis

**Figure 4.**
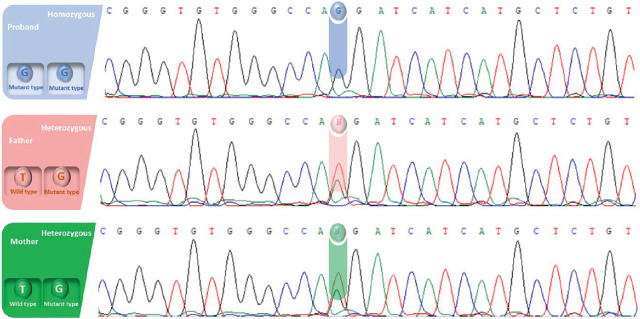
Sanger sequencing chromatograms confirm the presence of a novel homozygous variant (c.1274T>G; p.Met425Arg) in the *OCA2* gene in the proband. Both parents are heterozygous carriers, consistent with autosomal recessive inheritance.

The *OCA2* variant NM_000275.3:c.1274T>G (p.Met425Arg) was validated using Sanger sequencing in the proband and his consanguineous parents. The results confirmed that the proband was homozygous for the variant, while both parents were heterozygous carriers (**Figure 4**). This segregation pattern aligns with an autosomal recessive inheritance mode, consistent with the genetic etiology of OCA2.

PCR and sequencing chromatograms showed a clear nucleotide substitution (T>G) in the proband, while the parents’ chromatograms revealed overlapping peaks corresponding to both wild-type (T) and mutant (G) alleles. These findings confirm the familial transmission of the variant and its correlation with the observed phenotype.

### Genetic counseling for family planning

The identification of a novel variant in the *OCA2* gene highlights the importance of genetic counseling for families affected by albinism. In this case, the consanguineous nature of the parents increases the risk of autosomal recessive disorders in offspring. Genetic counseling can inform the family about recurrence risks and provide education on the inheritance patterns of *OCA2*-related conditions.

### Prenatal and perinatal guidance

Prenatal and perinatal guidance is essential for families carrying a confirmed genetic variant, such as *OCA2*, c.1274T>G; p.Met425Arg. Options include targeted genetic testing through procedures like chorionic villus sampling (CVS) or amniocentesis to determine the genetic status of the fetus. Early identification of affected pregnancies can assist in preparing for specialized care and management at birth.

### Reproductive technologies

The identification of the c.1274T>G; p.Met425Arg variant in the *OCA2* gene provides a foundation for the application of advanced reproductive technologies to mitigate the recurrence risk of albinism in future generations. In vitro fertilization (IVF), combined with preimplantation genetic diagnosis (PGD), offers a precise method to screen embryos for the identified pathogenic variant. Through PGD, embryos can be evaluated for the presence of this variant before implantation, enabling the selection of unaffected embryos.

## Discussion

The identification of the novel c.1274T>G (chr15:g.27985154 A>C, GRCh38/hg38) variant in the *OCA2* gene, located in exon 13 and resulting in a methionine-to-arginine substitution at position 425 (p.Met425Arg), highlights a potential molecular mechanism underlying the patient’s OCA2. Methionine, a non-polar amino acid, is replaced by arginine, a positively charged and polar residue [14,15,16], which alters the protein’s local environment, likely disrupting its stability and function. The pathogenicity of this variant is supported by the high conservation of the residue across species, indicating its functional importance. Segregation analysis confirms autosomal recessive inheritance, with the proband homozygous for the variant and both consanguineous parents heterozygous carriers. These findings provide a comprehensive understanding of how this amino acid change may impair OCA2 function, resulting in the observed phenotype, which includes OCA features such as hypopigmentation, strabismus, and photosensitivity.

The novel variant identified in the *OCA2* gene (c.1274T>G; p.Met425Arg) further underscores the critical role of OCA2 in melanin synthesis and pigmentation. The OCA2 protein is a transmembrane protein localized to the melanosome membrane, essential for regulating the transport of ions and other molecules necessary for the synthesis and stabilization of melanin precursors [17]. Disruptions in OCA2 function caused by the p.Met425Arg substitution likely impair melanosomal pH balance and tyrosinase activity, the rate-limiting enzyme in melanin biosynthesis [6,7,8]. This biochemical disruption provides a plausible explanation for the hypopigmentation observed in the patient. Interestingly, the Met425 residue lies in a conserved region of OCA2 that may contribute to its structural integrity or interaction with other proteins involved in melanin synthesis. Structural modeling suggests that the substitution of methionine with arginine introduces a charged residue into a hydrophobic environment, destabilizing the protein and impairing its integration into the melanosome membrane. These effects could disrupt the protein’s function, emphasizing the pathogenic nature of the variant. Moreover, this finding contributes to the growing body of evidence linking specific *OCA2* variants to the clinical variability observed in *OCA2*-related albinism. While most patients exhibit classic signs of albinism, such as hypopigmentation and visual abnormalities, there can be variability in the severity of these symptoms, influenced by genetic modifiers or environmental factors [18, 19]. The proband’s consanguineous parents being heterozygous carriers without any clinical manifestations further supports the autosomal recessive inheritance of this disorder, aligning with established genetic patterns of *OCA2* variants.

The newly c.1274T>G; p.Met425Arg variant detected in this study represents a significant addition to the expanding catalog of *OCA2* variants, highlighting a unique structural and functional disruption within the OCA2 protein. As discussed earlier, after establishing the biochemical rationale and inheritance pattern of this variant, this section emphasizes its broader implications for understanding the OCA phenotype. The introduction of a positively charged residue in a highly conserved region likely alters not only the local protein conformation but also critical interactions with melanosomal partners, such as TYR and other enzymatic components essential for melanin biosynthesis [3, 5]. This mechanistic disruption aligns with the consistent hypopigmentation and photosensitivity observed in our patient. Similar comparisons can be drawn with other pathogenic *OCA2* variants (**Table 1**), such as p.Pro198Leu and p.Asn476Asp, which cause severe hypopigmentation but vary in phenotype depending on population-specific modifiers. For instance, variants like p.Ser820Pro in Pakistani patients or p.Arg137Ilefs*83 are linked to overlapping features, such as nystagmus and photophobia, with additional hallmarks like foveal hypoplasia [20]. The newly detected variant parallels these pathogenic changes but provides further insight into the interplay between protein stability and melanosomal function, especially in regulating melanosomal pH and melanin synthesis.

**Table 1.** Summary of reported *OCA2* variants from previous studies

**No.**	**Variant(s)**	**Protein change(s)**	**Type of variant(s)**	**ACMG**	**Clinical findings**	**Population**	**Reference**
**1**	c.593C>T, c.1426A>G	p.Pro198Leu, p.Asn476Asp	Missense, missense (compound heterozygous)	Pathogenic, likely pathogenic	Milky white skin, blond hair, green irises, nystagmus	Chinese	Wang et al. [21]
**2**	c.2458T>C	p.Ser820Pro	Missense (homozygous)	VUS	White hair, pale skin, nystagmus, photophobia, and foveal hypoplasia	Pakistani	Arshad et al. [20]
**3**	c.408_409delAA	p.Arg137Ilefs*83	Frameshift (heterozygous)	Pathogenic	White hair, pale skin, nystagmus, photophobia, and foveal hypoplasia	Pakistani	Arshad et al. [20]
**4**	c.1762C>T	p.Arg588Trp	Missense (heterozygous)	Benign	White hair, pale skin, nystagmus, photophobia, and foveal hypoplasia	Pakistani	Arshad et al. [20]
**5**	c.1045-15T>G	—	Splicing (homozygous)	Likely pathogenic	White hair, pale skin, nystagmus, photophobia, and foveal hypoplasia	Pakistani	Arshad et al. [20]
**6**	c.2020C>G	p.Leu674Val	Missense (homozygous)	Likely pathogenic	White hair, pale skin, nystagmus, photophobia, and foveal hypoplasia	Pakistani	Arshad et al. [20]; Lee et al. [22]
**7**	c.1327G>A	p.Val443Ile	Missense (homozygous)	Likely pathogenic	White hair, pale skin, nystagmus, photophobia, and foveal hypoplasia	Pakistani	Arshad et al. [20]; Mondal et al. [23]

ACMG, American College of Medical Genetics and Genomics.

The identified novel homozygous missense variant in the *OCA2* gene, c.1274T>G (p.Met425Arg), falls within the predicted transmembrane domain (amino acids 424–440) of the OCA2 protein, which is essential for its structural and functional integrity. This domain has been implicated in various pathogenic variants associated with OCA. Notably, the p.Met425Arg variant was predicted by MutationTaster to result in the loss of the transmembrane domain, which is consistent with the patient’s phenotype of OCA features, including white hair, blue eyes, strabismus, and sun-sensitive skin. Similar genotype–phenotype correlations have been reported for other pathogenic variants within or near transmembrane domains of OCA2. For instance, the p.Val443Ile (c.1327G>A) variant, located close to the p.Met425Arg variant, has been associated with typical albinism features and classified as likely pathogenic [20, 23]. Additionally, other variants, such as p.Leu674Val (c.2020C>G), have shown similar phenotypic presentations, including nystagmus, photophobia, and foveal hypoplasia [20, 22]. These findings underscore the critical role of the transmembrane domains in OCA2’s function and support the likely pathogenicity of the detected variant in this consanguineous Iranian family.

The high conservation of methionine at position 425 across species underscores the evolutionary importance of this residue in maintaining OCA2 functionality. In contrast, variants in other regions, such as p.Gly780Ser, may result in milder phenotypes, suggesting variable tolerance to amino acid substitutions based on structural context [24]. Importantly, our findings support the hypothesis that phenotypic severity in *OCA2*-related albinism depends not only on the type and location of variants but also on potential genetic modifiers, environmental factors, and residual protein activity.

Genetic modifiers significantly influence the phenotypic variability observed in individuals with *OCA2* variants. One of the most studied modifiers is the *MC1R* gene, which regulates the balance between eumelanin and pheomelanin synthesis. For example, loss-of-function variants such as p.R151C and p.R160W in *MC1R* are associated with red hair color and reduced pigmentation and have been shown to exacerbate hypopigmentation in individuals with *OCA2* variants [25, 26]. Beyond *MC1R*, other genes such as *TYR* and *SLC45A2* act as genetic modifiers. Variants like p.S192Y in *TYR* may affect residual enzymatic activity, while p.L374F in *SLC45A2* has been linked to pigmentation differences in European populations [27,28,29,30]. For our patient, who carries the novel homozygous missense variant c.1274T>G (p.Met425Arg) in *OCA2*, investigating the co-segregation of these modifier variants could explain the specific phenotype observed, including white hair, blue eyes, and sun-sensitive skin. Including such analyses would enrich our understanding of genotype-phenotype interactions in albinism and highlight the role of genetic modifiers in phenotypic variability.

By documenting the likely pathogenic impact of c.1274T>G, this study underscores the critical need for comprehensive molecular and clinical characterization of *OCA2* variants. These findings refine genotype-phenotype correlations and enhance our understanding of how specific variants influence disease severity. Additionally, they inform genetic counseling strategies, especially in consanguineous populations where autosomal recessive inheritance patterns are prevalent. In line with our in silico pathogenicity predictions, which classified the p.Met425Arg variant as likely pathogenic based on multiple bioinformatics tools and ACMG criteria, this variant exemplifies how computational analyses can complement clinical assessments. The identification of p.Met425Arg strengthens the growing body of evidence that *OCA2* variants universally disrupt melanin production but exhibit diverse clinical manifestations shaped by complex biological and environmental interactions.

## Conclusion

This study identifies a novel *OCA2* variant, c.1274T>G (p.Met425Arg), that likely disrupts protein structure and melanosomal function, impairing melanin biosynthesis and resulting in OCA features. These findings contribute to the molecular and clinical characterization of *OCA2*-related albinism and expand the catalog of known *OCA2* variants. Further research is needed to investigate the molecular mechanisms underlying phenotypic variability, the role of genetic and environmental modifiers, and the impact of this variant through advanced functional studies. Addressing these gaps will refine genotype-phenotype correlations, enhance diagnostic precision, and improve genetic counseling and clinical management for affected populations.
